# Biomagnetic monitoring combined with support vector machine: a new opportunity for predicting particle-bound-heavy metals

**DOI:** 10.1038/s41598-020-65677-8

**Published:** 2020-05-25

**Authors:** Qian’ying Dai, Mengfan Zhou, Huiming Li, Xin Qian, Meng Yang, Fengying Li

**Affiliations:** 1 State Key Laboratory of Pollution Control and Resources Reuse, School of the Environment, Nanjing University, Nanjing, 210023 China; 2 School of Environment, Nanjing Normal University, Nanjing, 210023 China; 3 Jiangsu Collaborative Innovation Center of Atmospheric Environment and Equipment Technology (CICAEET), Nanjing University of Information Science & Technology, Nanjing, 210044 China; 4 Jiangsu Key Laboratory of Atmospheric Environment Monitoring and Pollution Control, School of Environmental Science and Engineering, Nanjing University of Information Science & Technology, Nanjing, 210044 China

**Keywords:** Atmospheric chemistry, Geomagnetism, Statistics

## Abstract

Biomagnetic monitoring includes fast and simple methods to estimate airborne heavy metals. Leaves of *Osmanthus fragrans Lour* and *Ligustrum lucidum Ait* were collected simultaneously with PM_10_ from a mega-city of China during one year. Magnetic properties of leaves and metal concentrations in PM_10_ were analyzed. Metal concentrations were estimated using leaf magnetic properties and meteorological factors as input variables in support vector machine (SVM) models. The mean concentrations of many metals were highest in winter and lowest in summer. Hazard index for potentially toxic metals was 5.77, a level considered unsafe. The combined carcinogenic risk was higher than precautionary value (10^−4^). Ferrimagnetic minerals were dominant magnetic minerals in leaves. Principal component analysis indicated iron & steel industry and soil dust were the common sources for many metals and magnetic minerals on leaves. However, the poor simulation results obtained with multiple linear regression confirmed strong nonlinear relationships between metal concentrations and leaf magnetic properties. SVM models including leaf magnetic variables as inputs yielded better simulation results for all elements. Simulations were promising for Ti, Cd and Zn, whereas relatively poor for Ni. Our study demonstrates the feasibility of prediction of airborne heavy metals based on biomagnetic monitoring of tree leaves.

## Introduction

Atmospheric particulate matter (PM) pollution is a serious global environmental problem and it has been closely linked to a wide range of adverse health outcomes^1–4^. Besides the size and concentration of PM, there is growing evidence that its chemical composition, especially its trace metal components, is crucial to its toxicity^5–7^. The metal particles adsorbed to PM can be transported over large distances and deposited in soils and water bodies or on the leaves of plants via wet and dry deposition, thereby posing environmental risks^8,9^. Consequently, there is an urgent need to investigate the pollution levels caused by particle-bound heavy metals in urban areas.

Tree leaves have large surface areas and thus serve as efficient passive collectors of atmospheric dust^10^. In general, coarse PM is usually deposited on leaf surfaces whereas finer PM is trapped in leaf waxes^11^. With their wide distribution in urban areas and ease of sampling, tree leaves have been used to investigate the spatial and temporal patterns of atmospheric pollutants, including PM^12,13^, heavy metals^10,14,15^, polycyclic aromatic hydrocarbons^16,17^, and nitrogen oxides^18,19^. Geochemical methods for determinations of atmospheric heavy metals involve the collection and processing of PM filter samples. Alternative methods include atomic absorption spectrometry, inductively coupled plasma atomic emission spectroscopy and ICP mass spectrometry, but they are laborious and time-consuming and therefore not suited to large-scale pollution monitoring. However, the magnetic particles adsorbed on tree leaves are a good proxy to measure pollution caused atmospheric heavy metals. Several reports have focused on the relationships between leaf magnetic properties and metal concentrations in leaf samples^10,20–23^ or in deposited atmospheric dust^23,24^. However, their methods cannot be used to directly analyze actual pollution caused by particle-bound heavy metals in the atmosphere, because metal concentrations are normally measured using PM of a certain particle size and collected on pumped-air filters. By contrast, few studies have evaluated the statistical relationships between leaf magnetic properties and heavy metals trapped on pumped-air filters^14^.

The levels of atmospheric pollutants can be predicted using deterministic and statistical models. Deterministic models usually require information about pollution sources and emission quantity, as well as sufficient knowledge about the physical processes and chemical reactions among pollutants^25,26^. Statistical models are simpler and better-suited to identifying the dependencies underlying pollutant concentrations and their potential predictors; as such, they often have a higher accuracy^27,28^. Among the statistical approaches used to forecast atmospheric pollutant levels are multiple linear regression^29,30^, grey model^31^, clusterwise regression^32^, random forest partition model^33^, artificial neural networks^34,35^, support vector machine^36,37^ and hybrid models^38,39^. Among those methods, the support vector machine (SVM) algorithm, which is based on the structural risk minimization principle, has increasingly been applied to solve non-linear regression problems, because it takes into account the error approximation in the data and generalization of the models. Previous studies used SVM approaches to predict a series of atmospheric pollutants, including NO_2_^40^, CO^41^ (GarcíaNieto *et al*., 2013), O_3_^42^ (Ortiz-García *et al*., 2010), PM (Cheng *et al*., 2019) and particle-bound heavy metals^43^, based on emission information and meteorological data. However, the potential of statistical models combined with leaf magnetic properties to predict atmospheric heavy metals has yet to be fully explored.

In this study, we examined the relationship between leaf magnetic characteristics and heavy metal concentrations in atmospheric PM_10_ from a large metropolitan city in China. Based on this relationship, we developed a method that uses leaf magnetic properties and meteorological factors as input variables in non-linear statistical models to accurately predict atmospheric particle-bound heavy metals.

## Results and Discussion

### **PM**_**10**_**concentrations**

**Figure 1:**
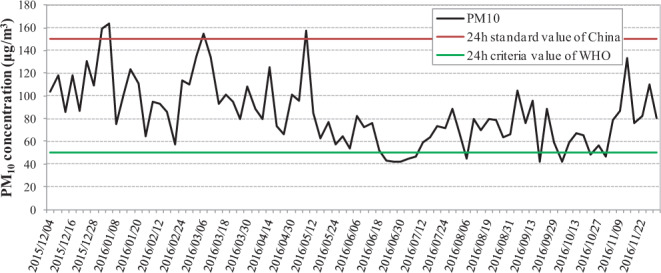
Trend in the 32-hour averaged PM_10_ concentrations (µg/m^3^) during the considered sampling period.

As shown in Fig. 1, approximately 88% of the daily PM_10_ concentrations were higher than the 24-h guideline value of 50 μg/m^3^ proposed by the World Health Organization (WHO), whereas <5% of the daily PM_10_ concentrations were above the 24-h Chinese National Ambient Air Quality Standard (NAAQS) limit of 150 μg/m^3^. The annual mean PM_10_ concentration in the study area was 84 μg/m^3^ (range: 42–164 μg/m^3^), which was slightly higher than the annual limit of 70 μg/m^3^ set by the NAAQS and much higher than the annual guideline value of 20 µg/m^3^ proposed by the WHO. The mean PM_10_ concentration changed seasonally, decreasing in the order of 105 μg/m^3^ (range: 58–164 μg/m^3^) in winter, 97 μg/m^3^ (range: 57–157 μg/m^3^) in spring, 75 μg/m^3^ in autumn (range: 42–133 μg/m^3^), and 64 μg/m^3^ in summer (range: 42–88 μg/m^3^). The temporal trends of the meteorological parameters and atmospheric pollutants during the sampling period are shown in Supplementary Figs. S1 and S2. The concentrations of atmospheric pollutants including PM_10_, PM_2.5_, SO_2_, NO_2_ and CO were higher in winter mainly because of the emissions of domestic heating systems and the unfavorable meteorological conditions, such as low wind speed and low temperature, which enhance the accumulation of air pollutants^44^. The lower concentrations of atmospheric pollutants during summer were related with the high temperature, abundant rain and the relatively strong diffusion capacity^45^.

### Concentrations of particle-bound-heavy metals

The mean concentrations of the particulate-bound elements are summarized in Table 1. In general, Fe and Zn were the most abundant heavy metals in PM_10_, whereas Co and Cd were present at lower concentrations. The mean concentrations of Cr, Cu, Fe, Mn, Pb and Zn were highest in winter, those of Cd, Ni and V were highest in autumn, and those of As, Co and Ti were highest in spring. The mean concentrations of most of the measured elements (except As and Co) were lowest in summer.

**Table 1:** Heavy metal concentrations in PM_10_ samples during the four seasons (ng/m^3^).

	Spring	Summer	Autumn	Winter	Average
As	8.54 ± 4.43	8.00 ± 3.87	6.43 ± 2.49	7.22 ± 3.73	7.57 ± 3.73
Cd	1.73 ± 0.77	1.52 ± 0.63	3.51 ± 1.04	2.17 ± 1.08	2.20 ± 1.17
Co	0.58 ± 0.23	0.49 ± 0.24	0.40 ± 0.20	0.48 ± 0.28	0.48 ± 0.25
Cr	38.4 ± 17.7	29.4 ± 10.8	39.0 ± 13.1	49.9 ± 14.1	38.8 ± 15.7
Cu	39.3 ± 18.4	29.9 ± 8.29	35.8 ± 16.5	46.8 ± 12.4	37.7 ± 15.4
Fe	1043 ± 453	689 ± 225	786 ± 420	1064 ± 284	891 ± 382
Mn	52.3 ± 26.1	35.9 ± 9.59	56.7 ± 16.3	62.0 ± 19.0	51.1 ± 20.8
Ni	54.8 ± 18.9	52.8 ± 21.2	82.1 ± 27.4	68.1 ± 23.9	63.8 ± 24.8
Pb	38.8 ± 15.5	30.9 ± 9.56	45.0 ± 14.0	62.0 ± 20.5	43.7 ± 18.8
Ti	52.1 ± 25.1	37.5 ± 16.4	41.7 ± 17.2	51.4 ± 16.4	45.5 ± 19.8
V	4.85 ± 1.83	4.55 ± 1.41	6.56 ± 2.77	4.74 ± 1.28	5.15 ± 2.14
Zn	296 ± 135	247 ± 125	355 ± 111	399 ± 102	321 ± 131

**Figure 2:**
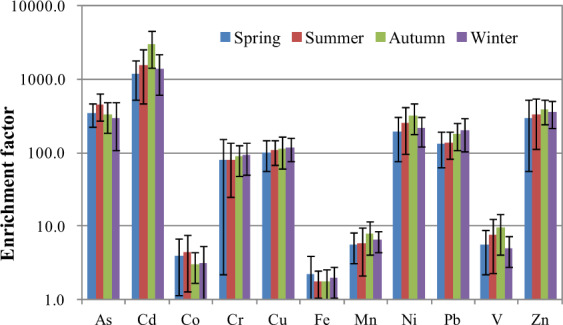
Enrichment factors of heavy metals in PM_10_ during the four seasons.

In assessments of heavy metal contaminations, the enrichment factor (EF), calculated by normalizing a tested element against a conservative reference element, is commonly used to distinguish between anthropogenic influences and natural background levels^46,47^. The calculation and classification of the EF as applied in this study can be found in the Supplementary Information. As shown in Fig. 2, there were no obvious seasonal difference in the EFs of the different elements. The mean EFs of Co, Fe, Mn, and V were <10, indicative of a minimal enrichment of these metals and their having originated mainly from crustal sources. Cr was moderately enriched (10 < EF < 100), whereas As, Cd, Cu, Ni, Pb and Zn (EF > 100) were anomalously enriched. Thus, all of these elements were likely to have derived from anthropogenic sources, such as steel smelting, fly ash from coal burning, vehicle emissions, waste incineration, and contaminated soil^48^.

### Health impacts based on guideline values and risk assessment

Comparisons of the elemental concentrations in PM_10_ with the limits imposed by the NAAQS (GB3095–2012) and WHO are shown in Supplementary Fig. S3. The Mn, Pb and V concentrations in all PM_10_ samples were far below the NAAQS (GB3095–2012) and WHO limits. The Cd concentration was lower than the NAAQS (GB3095–2012) and WHO limits with exception of three PM_10_ samples collected in autumn, in which the concentration was slightly higher. By contrast, the As concentration in 64.3% and 53.6% of the PM_10_ samples exceeded the NAAQS limit of 6 ng/m^3^ and 6.6 ng/m^3^, respectively. The Ni concentration in nearly all of the PM_10_ samples was above the WHO limit of 25 ng/m^3^.

The health risk caused by exposure to the analyzed airborne metals via inhalation is shown in Supplementary Table S1. Among the studied metals, Ni, Pb and As had higher EC values. The HQ values for the inhalation of As, Cd, Co, Cr, Mn and V were below the safe limit (1) for both children and adults. Ni had the highest HQ value (3.83) in these two subpopulations. The hazard index (HI) for these metals was 5.77, which was above the safe limit (1), indicating accumulative noncarcinogenic risks for adults and children. For carcinogens, the acceptable risk range is between 1 × 10^−6^ and 1×10^−4^ according to the US EPA’s risk management policy. The carcinogenic risks of As, Cd, Co, Ni, and Pb inhaled from PM_10_ were less than the precautionary value (10^−4^), both for children and adults, but the carcinogenic risks of Cr for the two subpopulations were higher. The combined carcinogenic risk was 2.39 × 10^−4^ for children and 9.55 × 10^−4^ for adults. Both values were higher than the precautionary value. Thus, for every one million children and one million adults living in the local environment, approximately 3 children and 10 adults are at risk of developing cancer during their lifetime due to exposure to toxic metals via PM_10_ inhalation.

### Leaf magnetic properties

**Figure 3:**
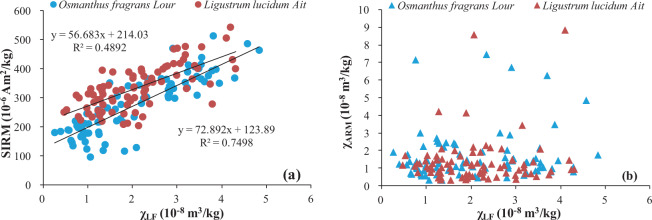
Scatter plots of (**a**) χ_LF_ vs. SIRM and (**b**) χ_LF_ vs. χ_ARM_ in the leaves of *Osmanthus fragrans Lour* and *Ligustrum lucidum Ait*.

Both χ_LF_ and SIRM generally reflect the quantities of magnetic, and especially ferromagnetic (e.g., magnetite) minerals in a sample, but they are also influenced by the presence of paramagnetic and diamagnetic minerals^49^. χ_ARM_ is particularly sensitive to single-domain ferrimagnetic grains^50^. As shown in Table 2, in the leaves of both tree species χ_LF_ and SIRM decreased seasonally, in the order of winter > spring > autumn > summer. However, there were differences in the seasonal pattern of χ_ARM_, which decreased in the order of winter > autumn > spring> summer for *Osmanthus fragrans Lour*, and in the order summer > winter > autumn > spring for *Ligustrum lucidum Ait*. As shown in Fig. 3, the SIRM and χLF values correlated linearly, suggesting that ferrimagnetic minerals were the dominant magnetic minerals in the leaf samples^48^. The lack of a significant correlation between χ_LF_ and χ_ARM_ (Fig. 3) indicated that single-domain grains did not dominate these ferrimagnetic minerals. The ratios of χ_ARM_ to χ_LF,_ χ_ARM_ to SIRM, and SIRM to χ_LF_ are used to estimate mineral magnetic grain-size variations, with increasing ratios indicating decreasing grain size^48,50,51^. For both tree species, all three ratios were lowest in spring and higher in autumn and summer, which suggested leaf accumulation of a larger number of finer grains in the latter two seasons.

**Table 2:** Magnetic properties of the collected leaf samples of *Osmanthus fragrans Lour* and *Ligustrum lucidum Ait*.

Property	*Osmanthus fragrans Lour*					*Ligustrum lucidum Ait*				
	Spring	Summer	Autumn	Winter	Average	Spring	Summer	Autumn	Winter	Average
χ_LF_ (10^–8^ m^3^/kg)	2.63 ± 0.93	0.88 ± 0.31	1.63 ± 0.88	3.27 ± 0.82	2.07 ± 1.19	2.36 ± 0.74	1.46 ± 0.46	1.57 ± 0.63	3.08 ± 0.80	2.10 ± 0.93
χ_ARM_ (10^–8^ m^3^/kg)	1.41 ± 1.49	1.06 ± 0.35	2.11 ± 1.36	2.13 ± 1.86	1.65 ± 1.41	0.68 ± 0.31	1.64 ± 1.69	1.55 ± 0.85	1.56 ± 1.84	1.36 ± 1.37
SIRM (10^–6^ Am^2^/kg)	289 ± 108	184 ± 32.1	248 ± 51.8	390 ± 54.1	274 ± 100	304 ± 60.5	275 ± 41.0	333 ± 38.4	431 ± 46.7	333 ± 75.0
χ_ARM_/χ_LF_	0.59 ± 0.67	1.51 ± 1.41	1.82 ± 2.00	0.66 ± 0.56	1.15 ± 1.38	0.30 ± 0.12	1.20 ± 0.96	1.11 ± 0.69	0.49 ± 0.45	0.78 ± 0.75
χ_ARM_/SIRM (10^–5^m/A)	5.24 ± 4.84	5.83 ± 1.92	9.12 ± 7.04	5.33 ± 4.32	6.35 ± 4.97	2.28 ± 1.16	6.16 ± 6.78	4.75 ± 2.79	3.48 ± 3.63	4.22 ± 4.42
SIRM/χ_LF_ (10^2^A/m)	111 ± 29.1	240 ± 124	179 ± 63.0	127 ± 35.8	166 ± 90.3	136 ± 30.5	209 ± 81.8	247 ± 102	149 ± 43.1	186 ± 82.5

The SIRM values and the ratio of SIRM to χ_LF_ were significantly higher in *Ligustrum lucidum Ait* than in *Osmanthus fragrans Lour*, whereas the ratios of χ_ARM_ to χ_LF_ and χ_ARM_ to SIRM were significantly lower in *Ligustrum lucidum Ait* than in *Osmanthus fragrans Lour*. The annual mean values of χ_LF_ and SIRM for *Osmanthus fragrans Lour* were 2.07 ± 1.19 × 10^−8^ m^3^/kg and 274 ± 100 × 10^−6^ Am^2^/kg, respectively, and for *Ligustrum lucidum Ait* 2.10 ± 0.93 × 10^−8^ m^3^/kg and 333 ± 75.0 × 10^−6^ Am^2^/kg, respectively (Table 3). According to a review of Hofman *et al*.^52^ and a previous study by our group^14^, the SIRM values of the two tree species were in the middle range while the χ_LF_ values were lower than published values. Several different factors determine leaf magnetic properties. Particle accumulation by leaves is influenced by species-specific characteristics of the trees, such as phenology, growth status, leaf area density and leaf characteristics, e.g., wax layer properties, surface roughness and trichomes presence^53^. However, sampling height, the leaf exposure period, PM source distance and strength, as well as meteorological conditions, e.g., wind, rain, drought and seasonal dynamics, also play important roles^52^. Further work is needed to reveal the underlying relationships among meteorological conditions, airborne heavy metals from various sources and leaf properties.

**Table 3:** The method and input variables of the five developed models.

	Method	Input variables
Model I	SVM	PM_10_ + meteorological factors
Model II	MLR	PM_10_ + meteorological factors + magnetic parameters of *Osmanthus fragrans Lour*
Model III	MLR	PM_10_ + meteorological factors + magnetic parameters of *Ligustrum lucidum Ait*
Model IV	SVM	PM_10_ + meteorological factors + magnetic parameters of *Osmanthus fragrans Lour*
Model V	SVM	PM_10_ + meteorological factors + magnetic parameters of *Ligustrum lucidum Ait*

### Principal component analysis (PCA)

The relationships among heavy metals, PM_10_, meteorological factors and magnetic parameters were analyzed in a PCA (Supplementary Tables S2 and S3). For the leaf magnetic parameters of *Osmanthus fragrans Lour*, five factors, accounting for 76.041% of the total variance, were obtained. The first factor, accounting for 35.272% of the total variance, was dominated by Cr, Cu, Fe, Mn, Pb, Ti, Zn, PM_10_, temperature, pressure, χ_LF_ and SIRM, which indicated that the metal sources were the iron and steel industry and soil dust. Factor 2, accounting for 15.619% of the total variance, was dominated by Cd, Ni, V, wind speed, χ_ARM_, χ_ARM_/χ_LF_, χ_ARM_/SIRM, and SIRM/χ_LF_, indicating industrial activities that resulted in the release of magnetic minerals of a certain grain size. Factor 3 explained 11.765% of the total variance and was dominated by As and Co. Arsenic is a typical element associated with coal combustion^54^, whereas Co, with EF < 10, is indicative of crustal source. Therefore, this factor may reflect mixed sources of coal combustion and natural process. Factor 4, accounting for 8.998% of the total variance, was dominated by relative humidity, whereas factor 5, dominated by Co and Cu and representing 4.386% of the total variance, indicated traffic activities and road dust as the major sources^55^.

When the leaf magnetic parameters of *Ligustrum lucidum Ait* were included in the PCA, four similar factors, accounting for 71.397% of the total variance, were obtained, with the first, second, third, and fourth components explaining 33.612%, 16.626%, 12.013%, 9.147% of the variance, respectively. The four factors were dominated by Cr, Cu, Fe, Mn, Pb, Ti, Zn, PM_10_, temperature, relative humidity, pressure, χ_LF_ and SIRM (component 1); Cd, Ni, V, wind speed, χ_ARM_/χ_LF_ and SIRM/χ_LF_ (component 2); As and Co (component 3) and χ_ARM_ and χ_ARM_/SIRM (component 4).

### Simulation results and implications

**Figure 4:**
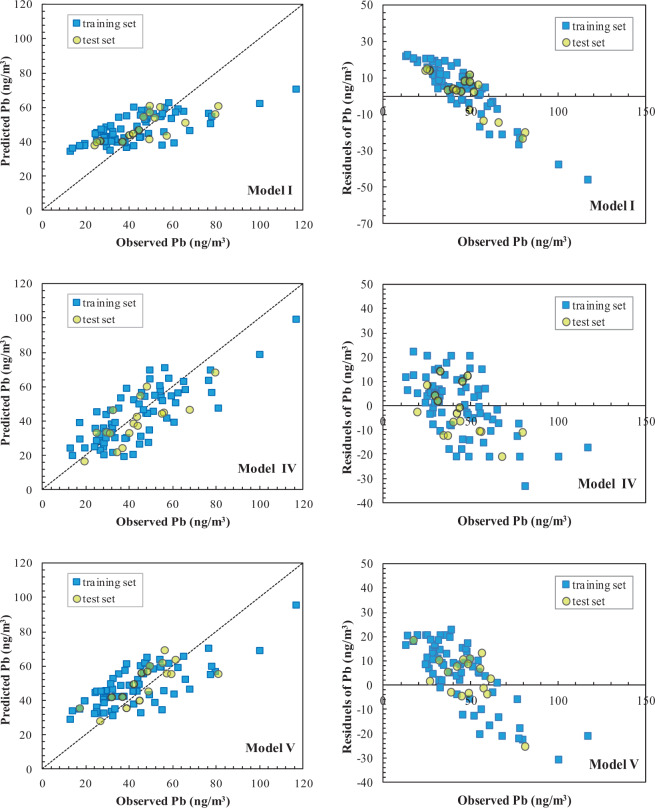
Predicted vs. observed concentrations and residuals plots of Pb for the training and test stages as described by models I, IV and V.

The linkage of atmospheric heavy metals and magnetic particles is based on the fact that heavy metals such as Zn, Cd, Pb and Cr can be incorporated into the particle structure during combustion processes and/or by subsequent surface adsorption^56,57^. Magnetic properties can thus act as an effective proxy for airborne heavy metals. The influence of leaf magnetic properties on heavy metal accumulation was examined in this study by predicting metal concentrations with and without leaf magnetic variables while including PM_10_ concentrations and meteorological factors (Table 3). The R, MAE and RMSE results are listed in Supplementary Tables S4–S7. The predicted vs. observed concentration and the residuals were plotted for Pb and are shown in Fig. 4.

When only the PM_10_ concentration and meteorological factors served as SVM inputs, the training R value of all the studied elements was between 0.565 and 0.819, and the test R value between 0.528 and 0.816. The training R and test R values of Cu were the lowest (0.565 and 0.528, respectively), and those of Ti the highest (0.819 and 0.816, respectively). The training R and test R values of Co, Cr, Fe, Mn and Ni were between 0.6 and 0.7, and those of As, Cd, Pb, V and Zn between 0.7 and 0.8.

However, the simulation results of the stepwise MLR were not satisfactory, even when leaf magnetic variables were included as input variables (Supplementary Table S5). For *Osmanthus fragrans Lour*, the training R values of the metals were between 0.587 (Mn) and 0.780 (Pb); for the test R, the values were <0.6, except in the case of Zn with test R of 0.693. For *Ligustrum lucidum Ait*, the training R values of all of metals were between 0.494 (Mn) and 0.681 (Pb); for the test R, the values were <0.6, with the exceptions of Cd and Co. These results obtained using a linear approach demonstrated the strong nonlinear relationships between metal concentrations and the input variables, which is consistent with our previous findings^30,43^.

When the leaf magnetic variables of *Osmanthus fragrans Lour* were included in the SVM model, the training R and test R values of all the elements were in the range of 0.693–0.918 and 0.667–0.903, respectively. The training and test R values of Cd, Cu, Ti and Zn were >0.8, with the highest values being those of Ti (0.918 and 0.903, respectively). Both the training and the test R values of As, Co, Cr, Fe, Mn, Pb and V were between 0.7 and 0.8 whereas Ni had the lowest values (0.693 and 0.667, respectively). The addition of the leaf magnetic variables of *Ligustrum lucidum Ait* into the SVM model yielded training R and test R values for all metals in the range of 0.661–0.875 and 0.630 to 0.859, respectively. The training and test R values of Cd, Ti, V and Zn were >0.8, with Ti again having the highest value (0.875 and 0.859, respectively). Both the training and the test R values of As, Co, Cu, Fe, Mn and Pb were between 0.7 and 0.8, but Cr and Ni had lower training and test R values (0.6–0.7). Thus, when the leaf magnetic variables were added as inputs, the training and the test R values of all the elements increased to varying degrees. The lower MAE and RMSE values of most of the metals (except Cr and Pb in model V) obtained in the training stage demonstrated the improved accuracy of model IV and model V when the leaf magnetic properties were included. In the test stage, the MAE and RMSE values of As, Cu, Mn, Pb, Ti, V and Zn of model IV, as well as As, Cd, Fe, Pb, Ti and V of model V were lower than the corresponding values of model I.

The improvement in the models achieved by including leaf magnetic properties as inputs was quantified by calculating the improvement rates (IRs) of model IV and model V for the training R and test R of each metal. The IR was calculated as follows^43^:

1 $${\rm{IR}}=({{\rm{R}}}_{{\rm{model}}{\rm{IV}}/{\rm{V}}}-{{\rm{R}}}_{{\rm{model}}{\rm{I}}})/{{\rm{R}}}_{{\rm{model}}{\rm{I}}}$$

**Figure 5:**
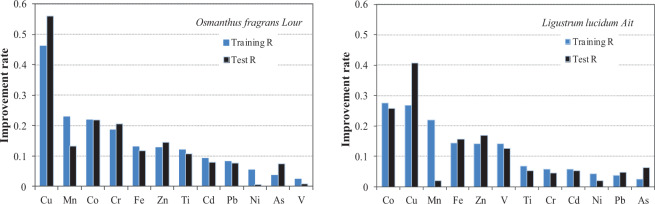
Improvement rate obtained by comparison of models IV (*Osmanthus fragrans Lour*) and I, and models V (*Ligustrum lucidum Ait*) and I.

As shown in Fig. 5, in the training stage, the IRs of Co, Cu and Mn were better whereas those of As, Ni and Pb were relatively poor, both for model IV and model V. In the test stage, the IR values of Cu, Co and Cr of model IV were higher, as were those of Cu, Co, Zn and Fe of model V (both models: >0.15). By contrast, the IR values of Ni and V of model IV and those of Mn, Ni, Cr and Pb of model V were lower (both models: <0.05). In general, Cu and Co, both of which were attributed in the PCA to traffic activities and road dust, improved by the inclusion of the leaf magnetic properties of *Osmanthus fragrans Lour* and *Ligustrum lucidum Ait*. In our previous report^43^, the IRs of Co, Cu, Fe, Mn and Zn were also better with the inclusion of magnetic variables of PM_2.5_ when simulating the mass-related concentrations of heavy metals in PM_2.5_ by using SVM models.

Model IV, in which the leaf magnetic properties of *Osmanthus fragrans Lour* were included as inputs, showed better simulation effects for As, Cd, Cr, Cu, Mn, Ni, Pb and Ti. For these metals, the training and test R values of this model were higher than those of model V (*Ligustrum lucidum Ait*), whereas the simulation of Co, Fe, V and Zn was better in model V. This result was mainly related to the morphological and physiological characteristics of the two tree species (e.g., leaf area density, wax layer properties, surface roughness)^58^ but the slight differences in the ambient environments (e.g., soil dust, road traffic, buildings) were also likely to have played a role^59^.

In general, models IV and V performed best for Ti, Cd and Zn, as evidenced by training R and test R values > 0.8. For Ni, however, the performances of these models were relatively poor, based on training R and test R values < 0.7. Ti was identified as a crustal element, whereas Ni, with the highest noncarcinogenic health risk and originating from mixed industrial activities, had a relatively poor simulation and very little improvement afforded by the inclusion of leaf magnetic properties. This finding is consistent with previous reports of a more reliable linkage between heavy metal concentrations and magnetic parameters in environments with similar and/or “single” source contributions, whereas multiple sources of heterogeneous chemical and magnetic particle can complicate determinations of the relationships between atmospheric heavy metals and magnetic parameters^52,60^. Source-specific magnetic fingerprints and their associations with atmospheric heavy metals remain to be elucidated in further research.

## Conclusions

The linkage between heavy metals in PM_10_ and the leaves of *Osmanthus fragrans Lour* and *Ligustrum lucidum AitLigustrum lucidum Ait*c were studied using SVM models. The annual mean PM_10_ concentration was 84 μg/m^3^ (range: 42–164 μg/m^3^). The elements As, Cd, Cu, Ni, Pb and Zn were anomalously enriched and Cr was moderately enriched whereas Co, Fe, Mn, and V were mainly from crustal sources. Ni had the highest noncarcinogenic risk, and Cr the highest carcinogenic risks. The combined noncarcinogenic and carcinogenic risks posed by inhalation exposure to airborne heavy metals were both above the safe limit or precautionary level. The χ_LF_ and SIRM values of the leaves of both tree species decreased in the order of winter > spring > autumn > summer, and the χ_ARM_ values in the order of winter > autumn > spring > summer. The dominant magnetic minerals in the leaf samples were ferrimagnetic minerals. PCA revealed that the heavy metals in PM_10_ have common sources with the magnetic minerals in leaf samples.

A subset of PM_10_ concentrations, meteorological factors and leaf magnetic properties were then used as input variables to simulate heavy metals concentrations. The poor simulation results obtained by MLR evidenced the nonlinear relationships between the airborne metal concentrations and the input variables. The inclusion of leaf magnetic variables improved the simulation results for all of the studied elements, with the largest improvements in Cu and Co and the lowest improvement in Ni. SVM models with leaf magnetic variables of the two tree species as inputs performed better for Ti, Cd and Zn but relatively poorly for Ni. Our study thus demonstrates that the concentrations of most airborne toxic heavy metals can be estimated using a simple and efficient biomagnetic diagnostic method.

## Methods

### Sampling

Nanjing (118°46′E, 32°03′N), the second largest city in the Yangtze River Delta region of China, is an important industrial production area and the main transportation hub in southeastern China. It has a north subtropical monsoon climate with a mean annual temperature of 16 °C and a mean annual precipitation of 1106 mm. PM_10_ samples were collected on Whatman quartz microfibers using medium-volume PM samplers (model XY-2200, Qingdao Xuyu Environmental Co., Ltd., China) with a flow rate of 100 L/min from the Xianlin Campus of Nanjing University (Supplementary Fig. S4), located in the northern suburbs of Nanjing and near the city’s northern industrial districts. Continuous sampling of PM_10_ lasted 32 h with 8 h (7:00 am-15:00 pm) per day was conducted, from December 4, 2015 to February 28, 2016 (winter), March 2 to May 28, 2016 (spring), June 2 to August 31, 2016 (summer) and September 4 to November 30, 2016 (autumn). To ensure the samples were representative, we avoided sampling during rainy or windy weather. A total of 84 PM_10_ samples were collected. Meteorological data were recorded synchronously at an automatic air quality monitoring station located near the study site. Before and after sampling, the filters were conditioned for 48 h in a desiccator at 25 °C and 40% relative humidity, and then weighed to determine PM_10_ mass.

*Osmanthus fragrans Lour* and *Ligustrum lucidum Ait*, two species of evergreen trees widely distributed in Nanjing, were selected for leaf sampling because the hairs on their leaf surfaces facilitate the adsorption of atmospheric particles. Leaf samples of the two trees were collected every 4 day during the same duration as PM sampling to keep consistence with the 32 h-sampled PM filters, from a site ~400 m away from the PM sampling site (Fig. S4). Specifically, each leaf sample was collected on the forth day of PM sampling to ensure the necessary accumulation of PM on leaves. The 4-day sampling duration both of PM_10_ and tree leaf was chosen by considering the effects of meteorological conditions and the daily variation of PM_10_ concentration from Nanjing. The distance between the two tree species was <100 m. For each tree species, two healthy trees next to each other were selected from which four leaves were collected from each one using ceramic scissors. The trees used for sampling in this study were all 3- to 4-year-old with a height of 2–3 m. The leaves were obtained from different sides of the tree and at a height from the ground of 1.5–2.0 m. The eight leaves were pooled to obtain one leaf sample, with 84 leaf samples collected in total for each tree species. The leaf samples were immediately placed in polyethylene bags and kept in a refrigerator at 4 °C. All leaf samples were totally dried in an oven at 55–60 °C before the analysis.

### Magnetic measurements

Low frequency (0.875 kHz; χ_LF_) was measured on about 2 g dried leaf sample using a KLY-3S kappa bridge (Agico, Czech Republic). Isothermal Remanent Magnetization (IRM) was induced in a field of 1000 mT (SIRM) using a Molspin pulse magnetizer. The anhysteretic remanent magnetization (ARM) was realized using a DTECH AF demagnetizer (Molspin, UK), delivering 0.04 mT of direct current (DC) and a peak alternating field of 100 mT. Measurements are then expressed as susceptibility of ARM (χ_ARM_) by dividing the remanence by the DC bias field. For the purpose of quantitation, all magnetic properties of each leaf sample were normalized on mass-specific basis.

### Analysis of heavy metal concentrations

Metal elements were released from the PM_10_ samples by digestion with a mixture of HNO_3_, HCl and HF. The concentrations of Fe and Zn were determined using inductively coupled plasma optical emission spectrometry (Perkin Elmer SCIEX, Optima 5300 DV, Norway). The concentrations of As, Cd, Co, Cr, Cu, Mn, Ni, Pb, Ti, V and Zn were determined by using inductively coupled plasma mass spectrometry (Perkin Elmer SCIEX, Elan 9000, Norway). Four blank filters were also digested and measured for metal concentrations simultaneously. Then the concentration of one element was corrected by subtracting its average concentration of blank filters. SRM 1649a (urban particulate matter) was used for quality assurance and control with the recovery of all the studied elements between 88% and 109%.

### Health risk assessment

The carcinogenic and noncarcinogenic risks posed by potentially toxic metals through the direct inhalation of PM_10_ were calculated using the human health risk assessment models of the US Environmental Protection Agency^61,62^. The models include exposure assessment and risk characterization. Sensitivity was determined for children and adults. The inhalation exposure concentration (EC), hazard quotient (HQ) of the noncarcinogenic risk, and the carcinogenic risk (CR) were calculated as described in the Supporting Information. The hazard index (HI) is equal to the sum of the HQ and was used to assess the overall potential of noncarcinogenic effects.

### Simulation models

Although initially developed for classification problems^63^, SVM models have been extended to solve nonlinear regression estimations by the introduction of an *ε*-insensitive loss function. Detailed information on SVM theory is provided in several publications^36,37,64^ and is thus described only briefly in the following:

Firstly, a kernel function is used to map the input variables to a high-dimensional feature space, after which the SVM approximates a set of data with a linear function:

2 $$y=f(x,\omega )=\mathop{\sum }\limits_{i=1}^{m}{\omega }_{i}\varnothing ({x}_{i})+b$$

where $\varnothing ({x}_{i})$ is the features of the input variables after their kernel transformation, and ${\omega }_{i}$ and b are the coefficients estimated by minimizing the regularized risk function. After kernel transformation, the data are linearly separable in the new feature space. In this study, the Gaussian radial basis function kernel was applied:

3 $$k({x}_{i},{x}_{j})=\exp (\,-\,\gamma \cdot ||{x}_{i}-{x}_{j}|{|}^{2})$$

where γ is the parameter of the kernel, and xi and xj are two independent variables.

The coefficients ${\omega }_{i}$ and b are estimated by minimizing the regularized risk function:

4 $$R=C\frac{1}{N}\mathop{\sum }\limits_{i=1}^{N}{L}_{\varepsilon }({y}_{i},f({x}_{i},\omega ))+\frac{1}{2}||\omega |{|}^{2}$$

where the term $C\frac{1}{N}{\sum }_{i=1}^{N}{L}_{\varepsilon }({y}_{i},f({x}_{i},\omega ))$ is the empirical error (risk), measured using the *ε*-insensitive loss function:

5 $${L}_{\varepsilon }({y}_{i},f({x}_{i},\omega ))=\{\begin{array}{ll}\,0, & \,if\,|y-f(x,\omega )|\le \varepsilon \\ |y-f(x,\omega )|-\varepsilon , & {\rm{otherwise}}\end{array}$$

where *ε* is a prescribed parameter called the regularized term and is defined as the approximation accuracy of the training data points. The loss function ignores errors when their value is less than that of *ε*. The term $1/2{{\rm{||}}\omega {\rm{||}}}^{2}$ is the regularization term, which serves as a measure of function flatness. The value of the regularized constant C determines the trade-off between empirical error and the regularization term. Finally, the dual problem of Eq. (4) is often resolved by the introduction of the Lagrange multiplier method:

6 $$f(x)=\mathop{\sum }\limits_{i=1}^{N}({\alpha }_{i}-{\alpha }_{i}^{\ast })K(x,{x}_{i})$$

where *α*_*i*_ and ${\alpha }_{i}^{\ast }$ are the introduced Lagrange multipliers.

In this study, MATLAB R2013a and libsvm-3.21 were used to build the SVM models. The data were randomly partitioned into two sets: 80% for training and 20% for testing. The maximum and minimum concentrations of one target element observed during the sampling period were retained in the training set to develop a reliable model. A subset of PM_10_ concentrations, meteorological factors (temperature, relative humidity, pressure, and wind speed), with or without leaf magnetic properties (χ_LF_, χ_ARM_, SIRM, χ_ARM_/χ_LF_, χ_ARM_/SIRM and SIRM/χ_LF_), were used as the input variables. Among the successful models, the best model was selected based on the higher correlation coefficient of the observed versus predicted output and on fewer errors in the training and test stages.

Models using a stepwise multiple linear regression (MLR) were also established to simulate metal concentrations with the same independent variables used in the SVM models, by applying SPSS 23.0. As shown in Table 3, five models were developed according to the statistical methods and input variables.

### Evaluation of model performance

The correlation coefficient (R) of the observed vs. predicted concentration of each heavy metal was used to measure the fit performance of each model. The mean absolute error (MAE) and root mean squared error (MSE), which provide a global estimate of the difference between the observed and predicted outputs, were used to measure residual errors. In general, a higher R combined with a lower MAE and RMSE was considered to indicate better modeling of a metal element. R, MAE and RMSE were calculated as described in the Supplementary Information.

## Supplementary information

Supplementary information.

Supplementary information.

## Data Availability

The data that support the findings of this study are available from the corresponding author upon reasonable request.
