# Supplementary material for: Biomagnetic monitoring combined with support vector machine: a new opportunity for predicting particle-bound-heavy metals
Source: Sci Rep. 2020 May 25;10:8605. doi: 10.1038/s41598-020-65677-8 (PMC7248096; doi:10.1038/s41598-020-65677-8)
Supplement: Supplementary file 1 — Supplementary information. [file 41598_2020_65677_MOESM1_ESM.docx]

**Biomagnetic monitoring combined with support vector machine: a new opportunity for predicting particle-bound-heavy metals**

Qian'ying Dai ^1^, Mengfan Zhou ^1^, Huiming Li ^2*^, Xin Qian ^1,3**^, Meng Yang ^3,4^ & Fengying Li ^3,4^

^1^ State Key Laboratory of Pollution Control and Resources Reuse, School of the Environment, Nanjing University, Nanjing 210023, China

^2^ School of Environment, Nanjing Normal University, Nanjing 210023, China

^3^ Jiangsu Collaborative Innovation Center of Atmospheric Environment and Equipment Technology (CICAEET), Nanjing University of Information Science & Technology, Nanjing 210044, China

^4^ Jiangsu Key Laboratory of Atmospheric Environment Monitoring and Pollution Control, School of Environmental Science and Engineering, Nanjing University of Information Science & Technology, Nanjing 210044, China

**Number of pages: 14**

**Number of figures: 4**

**Number of tables: 7**

**Figure S1.** Meteorological conditions (T=temperature, RH=relative humidity, WS=wind speed, P=pressure) during the sampling periods.

**Figure S2.** Atmospheric pollutants (PM_2.5_, SO_2_, NO_2_, CO and O_3_) during the sampling periods.

**Figure S3.** Comparison of heavy metal concentrations in PM_10_ to the Chinese National Ambient Air Quality Standard (NAAQS) (GB3095, 2012) and World Health Organization (WHO) limits during the sampling periods.

**
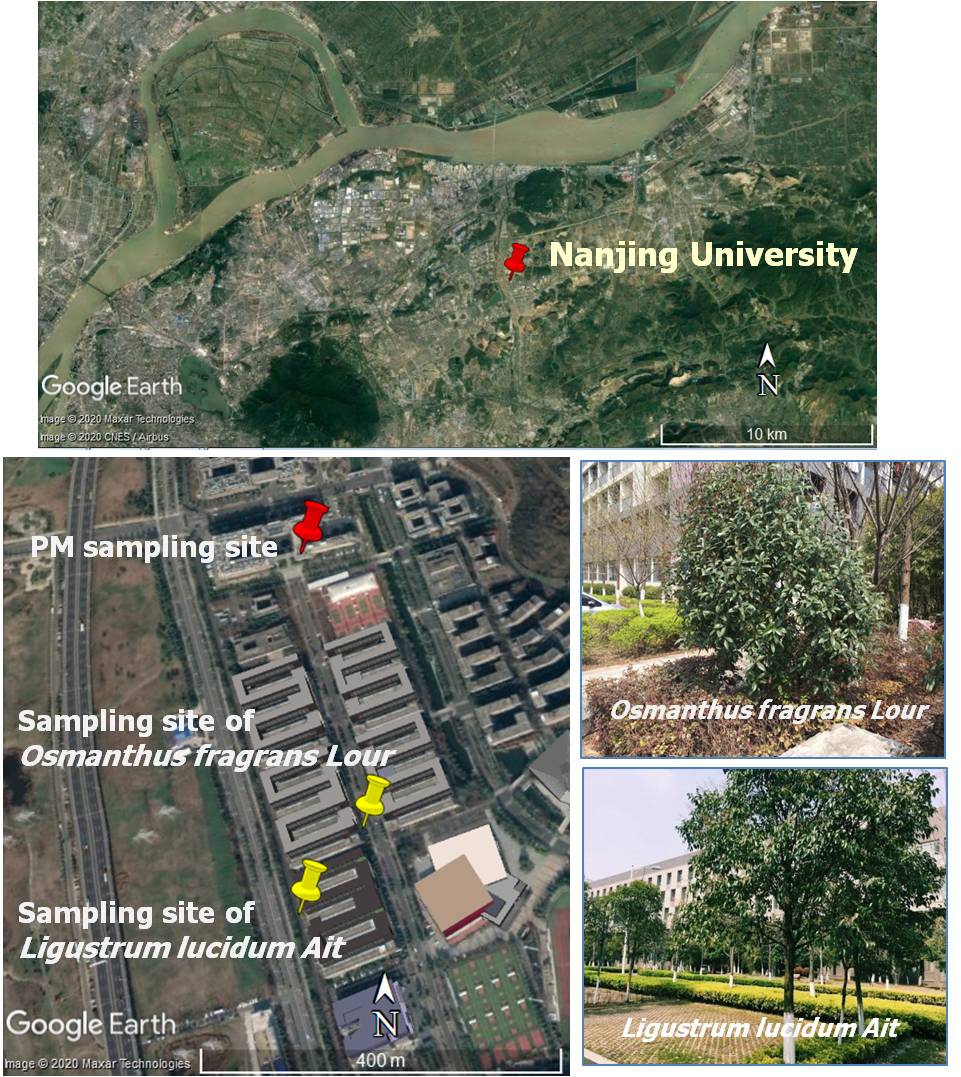
**

**Figure S4.** Sampling sites of particulate matter (PM) and tree leaves. (Satellite image from Maxar Technologies Inc. ; URL link: <https://www.earthol.com/>)

**Table S1.** The inhalation exposure concentration (EC) and the carcinogenic and noncarcinogenic risks due to inhalation exposure to the metal elements in PM_10_.

| Metal | CA(ng/m^3^) | IUR(ng/m^3^) | Rfc(ng/m^3^)^-1^ | EC(ng/m^3^) | | HQ | | CR | |
| --- | --- | --- | --- | --- | --- | --- | --- | --- | --- |
|  |  |  |  | Children | Adults | Children | Adults | Children | Adults |
| Non-carcinogenic |  |  |  |  |  |  |  |  |  |
| As(Inorganic) | 7.57 |  | 1.50E-05 | 6.37E+00 | 6.37E+00 | 4.25E-01 | 4.25E-01 |  |  |
| Cd(Diet water) | 2.53 |  | 1.00E-05 | 2.13E+00 | 2.13E+00 | 2.13E-01 | 2.13E-01 |  |  |
| Co | 0.48 |  | 6.00E-06 | 4.07E-01 | 4.07E-01 | 6.78E-02 | 6.78E-02 |  |  |
| Cr | 38.81 |  | 1.00E-04 | 3.26E+01 | 3.26E+01 | 3.26E-01 | 3.26E-01 |  |  |
| Mn(Diet) | 51.14 |  | 5.00E-05 | 4.30E+01 | 4.30E+01 | 8.61E-01 | 8.61E-01 |  |  |
| Ni(refinery dust) | 63.78 |  | 1.40E-05 | 5.37E+01 | 5.37E+01 | 3.83E+00 | 3.83E+00 |  |  |
| V | 5.13 |  | 1.00E-04 | 4.31E+00 | 4.31E+00 | 4.31E-02 | 4.31E-02 |  |  |
| Sum |  |  |  |  |  | 5.77E+00 | 5.77E+00 |  |  |
| Carcinogenic |  |  |  |  |  |  |  |  |  |
| As | 7.57 | 4.30E-03 |  | 5.46E-01 | 2.18E+00 |  |  | 2.35E-06 | 9.39E-06 |
| Cd | 2.53 | 1.80E-03 |  | 1.82E-01 | 7.28E-01 |  |  | 3.28E-07 | 1.31E-06 |
| Co | 0.48 | 9.00E-03 |  | 3.50E-02 | 1.39E-01 |  |  | 3.15E-07 | 1.25E-06 |
| Cr | 38.81 | 8.40E-02 |  | 2.80E+00 | 1.19E+01 |  |  | 2.35E-04 | 9.38E-04 |
| Ni(refinery dust) | 63.78 | 2.40E-04 |  | 4.60E+00 | 1.84E+01 |  |  | 1.10E-06 | 4.42E-06 |
| Pb(acetate) | 43.66 | 8.00E-05 |  | 3.15E+00 | 1.26E+01 |  |  | 2.51E-07 | 1.01E-06 |
| Sum |  |  |  |  |  |  |  | 2.39E-04 | 9.55E-04 |

**Table S2.** Principal component analysis*^a^* including heavy metal concentrations and the leaf magnetic parameters of *Osmanthus fragrans Lour* (PCA loadings > 0.5 are shown in bold).

| Factor | Component | | | | |
| --- | --- | --- | --- | --- | --- |
|  | 1 | 2 | 3 | 4 | 5 |
| As | 0.373 | -0.19 | **0.761** | 0.006 | 0.113 |
| Cd | 0.433 | **0.577** | -0.116 | 0.499 | 0.061 |
| Co | 0.355 | -0.143 | **0.607** | -0.222 | 0.405 |
| Cr | **0.765** | 0.153 | 0.047 | 0.047 | 0.258 |
| Cu | **0.734** | -0.054 | 0.151 | 0.093 | -0.419 |
| Fe | **0.734** | -0.063 | 0.252 | -0.044 | -0.009 |
| Mn | **0.748** | 0.225 | 0.087 | 0.106 | -0.299 |
| Ni | **0.516** | **0.611** | 0.237 | 0.134 | 0.019 |
| Pb | **0.807** | 0.216 | -0.117 | 0.103 | -0.065 |
| Ti | **0.663** | -0.082 | 0.441 | -0.203 | -0.113 |
| V | 0.364 | **0.528** | 0.281 | 0.432 | 0.301 |
| Zn | **0.731** | 0.404 | 0.023 | 0.097 | -0.257 |
| PM_10_ | **0.773** | -0.053 | 0.157 | -0.27 | 0.184 |
| WS | -0.421 | **0.604** | 0.009 | **0.504** | 0.054 |
| T | **-0.656** | 0.185 | **0.597** | 0.056 | 0.042 |
| RH | -0.49 | 0.218 | -0.225 | **0.511** | 0.207 |
| P | **0.617** | -0.056 | **-0.585** | -0.005 | 0.044 |
| χ_LF_ | **0.783** | -0.311 | -0.339 | -0.001 | 0.112 |
| SIRM | **0.824** | -0.069 | -0.377 | 0.017 | 0.011 |
| χ_ARM_ | 0.186 | **0.607** | -0.403 | **-0.529** | 0.213 |
| χ_ARM_/χ_LF_ | -0.251 | **0.764** | 0.079 | **-0.507** | -0.128 |
| χ_ARM_/SIRM | -0.145 | **0.703** | -0.175 | **-0.583** | 0.152 |
| SIRM/χ_LF_ | -0.42 | **0.504** | 0.276 | -0.115 | -0.377 |
| Initial eigenvalues | 8.113 | 3.592 | 2.706 | 2.069 | 1.009 |
| % of the variance | 35.272 | 15.619 | 11.765 | 8.998 | 4.386 |
| Cumulative % | 35.272 | 50.891 | 62.656 | 71.654 | 76.041 |

**Table S3.** Principal component analysis*^a^* including heavy metal concentrations and the leaf magnetic parameters of *Ligustrum lucidum Ait* (PCA loadings > 0.5 are shown in bold).

| Factor | Component | | | |
| --- | --- | --- | --- | --- |
|  | 1 | 2 | 3 | 4 |
| As | 0.378 | 0.075 | **0.698** | 0.259 |
| Cd | 0.394 | **0.621** | -0.215 | -0.403 |
| Co | 0.369 | -0.072 | **0.643** | 0.141 |
| Cr | **0.756** | 0.145 | 0.068 | -0.142 |
| Cu | **0.719** | 0.105 | 0.104 | 0.09 |
| Fe | **0.717** | 0.052 | 0.274 | 0.054 |
| Mn | **0.726** | 0.303 | 0.055 | -0.083 |
| Ni | **0.512** | **0.613** | 0.107 | -0.148 |
| Pb | **0.811** | 0.254 | -0.22 | 0.005 |
| Ti | **0.682** | 0.013 | 0.415 | 0.197 |
| V | 0.313 | **0.683** | 0.177 | -0.28 |
| Zn | **0.726** | 0.392 | -0.07 | -0.095 |
| PM_10_ | **0.784** | -0.063 | 0.172 | 0.193 |
| WS | -0.451 | **0.625** | -0.101 | -0.416 |
| T | **-0.675** | 0.309 | **0.524** | 0.137 |
| RH | **-0.509** | 0.289 | -0.325 | -0.224 |
| P | **0.632** | -0.197 | **-0.521** | -0.23 |
| χ_LF_ | **0.658** | -0.395 | -0.458 | 0.227 |
| SIRM | **0.697** | -0.116 | -0.453 | -0.049 |
| χ_ARM_ | 0.013 | **0.569** | -0.401 | **0.691** |
| χ_ARM_/χ_LF_ | -0.376 | **0.731** | -0.141 | 0.419 |
| χ_ARM_/SIRM | -0.197 | **0.573** | -0.305 | 0.71 |
| SIRM/χ_LF_ | -0.353 | **0.521** | 0.272 | -0.353 |
| Initial eigenvalues | 7.731 | 3.824 | 2.763 | 2.104 |
| % of variance | 33.612 | 16.626 | 12.013 | 9.147 |
| Cumulative % | 33.612 | 50.238 | 62.251 | 71.397 |

**Table S4.** Correlation coefficient (R), mean absolute error (MAE) and root mean squared error (RMSE) of model I.

| Metal | Training set | | |  | Test set | | |
| --- | --- | --- | --- | --- | --- | --- | --- |
|  | R | MAE | RMSE |  | R | MAE | RMSE |
| As | 0.709 | 2.267 | 2.771 |  | 0.715 | 2.128 | 2.414 |
| Cd | 0.779 | 0.701 | 0.841 |  | 0.798 | 0.446 | 0.542 |
| Co | 0.601 | 0.170 | 0.218 |  | 0.620 | 0.081 | 0.113 |
| Cr | 0.625 | 9.689 | 12.33 |  | 0.603 | 9.798 | 11.99 |
| Cu | 0.565 | 10.51 | 13.24 |  | 0.528 | 8.180 | 10.15 |
| Fe | 0.662 | 236.4 | 313.5 |  | 0.645 | 232.5 | 292.2 |
| Mn | 0.601 | 13.53 | 16.55 |  | 0.641 | 12.97 | 15.90 |
| Ni | 0.656 | 15.03 | 18.96 |  | 0.663 | 14.27 | 17.79 |
| Pb | 0.723 | 12.51 | 15.25 |  | 0.708 | 10.17 | 11.82 |
| Ti | 0.819 | 9.237 | 11.73 |  | 0.816 | 11.76 | 13.78 |
| V | 0.745 | 0.963 | 1.158 |  | 0.743 | 0.978 | 1.244 |
| Zn | 0.724 | 77.69 | 91.73 |  | 0.731 | 70.14 | 95.41 |

**Table S5.** Correlation coefficient (R) of the observed and predicted metal concentrations determined by multiple linear regression of models II and III.

| Metal | *Osmanthus fragrans Lour* | |  | *Ligustrum lucidum Ait* | |
| --- | --- | --- | --- | --- | --- |
|  | Training set | Test set |  | Training set | Test set |
| As | 0.660 | 0.384 |  | 0.618 | 0.552 |
| Cd | 0.677 | 0.371 |  | 0.636 | 0.684 |
| Co | 0.588 | 0.519 |  | 0.632 | 0.761 |
| Cr | 0.669 | 0.347 |  | 0.617 | 0.193 |
| Cu | 0.608 | 0.349 |  | 0.509 | 0.271 |
| Fe | 0.630 | 0.497 |  | 0.59 | 0.407 |
| Mn | 0.587 | 0.349 |  | 0.494 | 0.271 |
| Ni | 0.608 | 0.237 |  | 0.557 | 0.413 |
| Pb | 0.78 | 0.311 |  | 0.681 | 0.103 |
| Ti | 0.676 | 0.408 |  | 0.676 | 0.408 |
| V | 0.695 | 0.399 |  | 0.632 | 0.548 |
| Zn | 0.692 | 0.693 |  | 0.641 | 0.167 |

**Table S6.** Correlation coefficient (R), mean absolute error (MAE) and root mean squared error (RMSE) of model IV.

| Metal | Training set | | |  | Test set | | |
| --- | --- | --- | --- | --- | --- | --- | --- |
|  | R | MAE | RMSE |  | R | MAE | RMSE |
| As | 0.735 | 2.260 | 2.687 |  | 0.768 | 1.958 | 2.295 |
| Cd | 0.852 | 0.547 | 0.638 |  | 0.861 | 0.470 | 0.570 |
| Co | 0.733 | 0.145 | 0.184 |  | 0.756 | 0.124 | 0.153 |
| Cr | 0.742 | 8.733 | 10.30 |  | 0.727 | 12.30 | 13.97 |
| Cu | 0.827 | 8.536 | 9.841 |  | 0.823 | 5.312 | 6.418 |
| Fe | 0.750 | 198.8 | 257.1 |  | 0.722 | 252.3 | 292.3 |
| Mn | 0.740 | 11.63 | 14.09 |  | 0.726 | 12.61 | 14.02 |
| Ni | 0.693 | 13.94 | 17.44 |  | 0.667 | 20.22 | 24.97 |
| Pb | 0.782 | 10.03 | 12.25 |  | 0.763 | 8.680 | 10.06 |
| Ti | 0.918 | 7.377 | 8.761 |  | 0.903 | 8.987 | 10.15 |
| V | 0.762 | 0.959 | 1.139 |  | 0.750 | 0.773 | 0.920 |
| Zn | 0.817 | 70.26 | 82.01 |  | 0.837 | 48.99 | 62.42 |

**Table S7.** Correlation coefficient (R), mean absolute error (MAE) and root mean squared error (RMSE) of model V.

| Metal | Training set | | |  | Test set | | |
| --- | --- | --- | --- | --- | --- | --- | --- |
|  | R | MAE | RMSE |  | R | MAE | RMSE |
| As | 0.725 | 2.140 | 2.615 |  | 0.760 | 1.618 | 2.407 |
| Cd | 0.823 | 0.529 | 0.699 |  | 0.839 | 0.372 | 0.491 |
| Co | 0.766 | 0.136 | 0.161 |  | 0.779 | 0.124 | 0.146 |
| Cr | 0.661 | 10.20 | 12.50 |  | 0.630 | 9.802 | 12.12 |
| Cu | 0.717 | 8.950 | 10.79 |  | 0.743 | 8.609 | 10.89 |
| Fe | 0.757 | 210.2 | 283.3 |  | 0.745 | 183.3 | 250.7 |
| Mn | 0.733 | 12.88 | 14.41 |  | 0.654 | 15.45 | 18.24 |
| Ni | 0.683 | 14.87 | 17.61 |  | 0.676 | 16.10 | 21.24 |
| Pb | 0.750 | 12.72 | 14.39 |  | 0.742 | 8.131 | 10.26 |
| Ti | 0.875 | 8.115 | 9.777 |  | 0.859 | 9.134 | 10.63 |
| V | 0.850 | 0.848 | 0.989 |  | 0.837 | 0.886 | 1.146 |
| Zn | 0.826 | 73.37 | 85.45 |  | 0.854 | 80.68 | 99.11 |

**Method of health risk assessment**

The inhalation exposure concentration (EC) and the hazard quotient (HQ) of the noncarcinogenic and carcinogenic risks (CR) were calculated as follows:

$EC=(C\times\mathrm{ET}\times\mathrm{EF}\times ED)/\mathrm{AT}_{n}$, (1)

$HQ=EC/(RfC\times1000 \mu g/mg)$ (2)

$CR=IUR\times\mathrm{EC}$ (3)


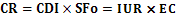


where ET is the exposure time (24 h/day), EF is the exposure frequency (180 days/year), ED is the exposure duration (6 years for children and 24 for adults), ATn is the average time (for non-carcinogens ATn = ED × 365 day × 24 h/day, and for carcinogens ATn = 70 year ×365 day/year × 24 h), RfC is the inhalation reference concentration (mg/m^3^), and IUR is the inhalation unit risk ((μg/m^3^)^-1^). The RfC, IUR, and standard default values for exposure parameters were taken from the user’s guide and technical background document for US EPA region 9, regional screening level tables^1^. The hazard index (HI) is equal to the sum of HQ and used to assess the overall potential noncarcinogenic effects. Cr(VI) and Cr(III) are classified as Group A (human carcinogens) and Group D (not classifiable for human carcinogenicity), respectively according to the US EPA^2^. The concentration ratio of Cr(VI) to Cr(III) in the atmosphere is reportedly about 1:6 ^3^, the concentration of Cr(VI) was then assumed to be one seventh of the total Cr concentration for the calculation of EC in this study.

**Calculation of indexes for model performance**

The correlation coefficient (R), mean absolute error (MAE), root mean squared error (RMSE), and index of agreement (IA) are described by Eqs.. (1–4), respectively:

$MAE=\frac{1}{n}\sum_{i=1}^{n} \left| \hat{Y}_{i}-Y_{i} \right|$ (4)

$RMSE=\sqrt{\frac{1}{n}\sum_{i=1}^{n} \left( Y_{i}-\hat{Y}_{i} \right)^{2}}$ (5)

$IA=1-\frac{\left[ \sum_{i=1}^{n} \left( \hat{Y}_{i}-Y_{i} \right)^{2} \right]}{\left[ \sum_{i=1}^{n} \left( \left| \hat{Y}_{i}-\bar{Y}_{i} \right|+\left| Y_{i}-\bar{Y}_{i} \right| \right)^{2} \right]}$ (6)

$R=\sqrt{\frac{\sum_{i=1}^{n} \left( Y_{i}-\bar{Y}_{i} \right)^{2}-\sum_{i=1}^{n} \left( Y_{i}-\hat{Y}_{i} \right)^{2}}{\sum_{i=1}^{n} \left( Y_{i}-\bar{Y}_{i} \right)^{2}}}$ (7)

where, $Y_{i}$ is the true target metric value for observation *i*, $\hat{Y}_{i}$ is the target metric value for observation *i* as predicted by the model, and *n* is the number of data.

**Calculation of enrichment factor (EF)**

EF values were calculated with respect to Ti as:

$EF={(C_{n}/C_{ref})}_{sample}/({B_{n}/B_{ref})}_{crust}$ (8)

where, ${(C_{n}/C_{ref})}_{sample}$ and $({B_{n}/B_{ref})}_{crust}$ ${(B_{n}/B_{\mathrm{ref}} )}_{\mathrm{baseline}}$are the concentration ratios of the target metal to the reference element Ti in PM_10_ samples and in the continental crust^4^, respectively. In this study, EF < 10 indicated that the target element was not enriched. EF > 10 indicated that the element derived from anthropogenic sources. In general, an EF < 10 indicates that the test element is minimally enriched, and an EF >10 that the element has anthropogenic sources. For 10 < EF < 100, the test elements are moderately enriched, while EF > 100 indicates anomalous enrichment.

**References**

1. US EPA (U.S. Environmental Protection Agency). User's Guide/technical Background Document for US EPA Region 9's RSL (Regional Screening Levels) Tables (2013). http://www.epa.gov/region9/superfund/prg/.

2. US EPA (U.S. Environmental Protection Agency). Risk Assessment Guidance for Superfund (RAGS), Volume I Human Health Evaluation Manual (Part F, Supplemental Guidance for Inhalation Risk Assessment). EPA-540-R-070e002, OSWER 9285.7-82, January (2009). http://www.epa.gov/swerrims/riskassessment/ragsf/index.htm.

3. Taner, S., Pekey, B. & Pekey, H. Fine particulate matter in the indoor air of barbeque restaurants: elemental compositions, sources and health risks. *Sci. Total Environ.* 454-455, 79-87 (2013).

4. Taylor, S.R. & Mclennan, S.M.. The geochemical evolution of the continental crust. *Rev. Geophys.* **33,** 241−265 (1995).
