# Supplementary material for: Biomagnetic monitoring combined with support vector machine: a new opportunity for predicting particle-bound-heavy metals
Source: Sci Rep. 2020 May 25;10:8605. doi: 10.1038/s41598-020-65677-8 (PMC7248096; doi:10.1038/s41598-020-65677-8)
Supplement: Supplementary file 2 — Supplementary information. [file 41598_2020_65677_MOESM2_ESM.docx]

**Figure S1.** Meteorological conditions (T=temperature, RH=relative humidity, WS=wind speed, P=pressure) during the sampling periods.

**Figure S2.** Atmospheric pollutants (PM_2.5_, SO_2_, NO_2_, CO and O_3_) during the sampling periods.

**Figure S3.** Comparison of heavy metal concentrations in PM_10_ to the Chinese National Ambient Air Quality Standard (NAAQS) (GB3095, 2012) and World Health Organization (WHO) limits during the sampling periods.

**Figure S4.** Sampling sites of particulate matter (PM) and tree leaves.

**Table S1.** The inhalation exposure concentration (EC) and the carcinogenic and noncarcinogenic risks due to inhalation exposure to the metal elements in PM_10._

**Table S2.** Principal component analysis*^a^* including heavy metal concentrations and the leaf magnetic parameters of *Osmanthus fragrans Lour* (PCA loadings > 0.5 are shown in bold).

**Table S3.** Principal component analysis*^a^* including heavy metal concentrations and the leaf magnetic parameters of *Ligustrum lucidum Ait* (PCA loadings > 0.5 are shown in bold).

**Table S4.** Correlation coefficient (R), mean absolute error (MAE) and root mean squared error (RMSE) of model I.

**Table S5.** Correlation coefficient (R) of the observed and predicted metal concentrations determined by multiple linear regression of models II and III.

**Table S6.** Correlation coefficient (R), mean absolute error (MAE) and root mean squared error (RMSE) of model IV.

**Table S7.** Correlation coefficient (R), mean absolute error (MAE) and root mean squared error (RMSE) of model V.
